# A new method for mutation inducing in rice by using DC electrophoresis bath and its mutagenic effects

**DOI:** 10.1038/s41598-023-33742-7

**Published:** 2023-04-25

**Authors:** Minmin Zou, Sun Tong, Ting Zou, Xinyi Wang, Linxuan Wu, Jiafeng Wang, Tao Guo, Wuming Xiao, Hui Wang, Ming Huang

**Affiliations:** Guangdong Provincial Key Laboratory of Plant Molecular Breeding, South China Agricultural University, Guangzhou, 510642 People’s Republic of China

**Keywords:** Genetics, Plant sciences

## Abstract

Mutation breeding is a significant means of increasing breeding efficiency and accelerating breeding process. In present study, we explored a new method for mutations inducing in rice (*Oryza sativa* L.) by using direct current electrophoresis bath (DCEB). The results showed that 20 mM NaCl solution is the optimal buffer, and the mortality of rice seeds followed an upward trend with increasing voltage and processing time of DCEB. By exploring the mutagenic effects of γ-irradiation and DCEB on seed vigor and physiological damages, we found that the physiological damages induced by DCEB on seed vigor were significant compared with that by γ-irradiation. We screened two mutants with low filled grain percentage and one mutant with abnormal hull from the M_2_ generations. These three mutants were confirmed to be authentic mutants based on 48 SSR markers followed by the protocol NY/T 1433–2014. Whole-genome resequencing detected a total of 503 and 537 polymorphisms in the two mutants, respectively, and the DCEB mutagenesis induced mainly InDel variants, while the exon region of mutant genes occupied a large proportion, especially the SNP variants, which occupied about 20% of the mutation sites in the exon region.

## Introduction

With the spread of the 2019 coronavirus disease (COVID-19), in the latest edition of The State of Food Security and Nutrition in the World 2020, the World Food and Agriculture Organization (FAO) predicted that the world will not reach the goal of zero hunger by 2030^1^, which means that the world will continue to face great challenges in food security. Rice (*Oryza sativa* L.) is the staple food for over half the world's population^2^. To ensure rice food security with changes in global climate and world population growth, there is an urgent need to develop high-yielding and high grain quality rice varieties at a greater pace^3,4^. Mutagenesis is a nearly 100-year-old technology, and mutation breeding currently plays a major role in the development of superior plant varieties worldwide^5^. However, spontaneous mutation rates for higher plants are low, ranging from 10^–5^ to 10^–8^, thus, mutagenesis is an important strategy to increase mutation frequencies^6^. The main mutagens are physical mutagens such as X-rays^7–9^, γ-irradiation^10–12^ and ion beams^13–16^, chemical mutagens such as ethyl methane sulfonate^17^ and sodium azide^18,19^. Scientists have developed rice varieties with various excellent traits, e.g. dwarfing, high yield and disease resistance^20,21^. Traditionally, mutations can be induced in a variety of ways, such as exposing plant propagules, including seeds, tissues and organs, to physical and chemical mutagens^22^. The most widely used method of mutation breeding, however, is the process of exposing seeds to chemical or physical mutagens to produce mutants that serve as new genetic resources with improved traits^23^. There are currently 3,365 registered mutant varieties in the International Atomic Energy Agency (FAO/IAEA) Mutant Variety Database, of which 2569 are seed propagated plants. Next-generation sequencing is increasingly being applied in single nucleotide polymorphism (SNP) detection and for assessing the effects of induced mutations^24^. Whole-genome resequencing (WGR) was employed to study the molecular characterization of mutations on a whole-genome level. Therefore, an increasing number of researchers have applied WGR technology to reveal mutagenic characteristics on the genome^25^.

Electrophoresis is a phenomenon in which charged molecules move in an electric field toward electrodes of opposite charge. The technique of using electrophoresis to separate substances is called electrophoretic technology, and an electrophoresis apparatus generally consists of a power supply, an electrophoresis bath, and a detection unit. In 1937, Tiselius, a Swedish scientist, designed the first electrophoresis apparatus, creating a new era of electrophoresis technology^26^. In recent years, electrophoresis has been widely used in various fields, such as biomedicine^27,28^, analytical chemistry^29^ and microbiology.

The present study provides a new method for mutations inducing in rice by using DC (direct current) electrophoresis bath (DCEB). Aimed to (1) explore the optimal treatment for DCEB mutagenesis; (2) compare the mutagenic effects induced by DCEB and by γ-irradiation, and (3) predict the mutation frequencies of DC electrophoretic bath mutagenesis by WGR. The present studies are presented as successful examples of this method which is simple, convenient, safe, flexible, and low costly in rice on mutation breeding.

## Results

### The optimal conditions for DC electrophoresis bath mutagenesis

**Figure 1:**
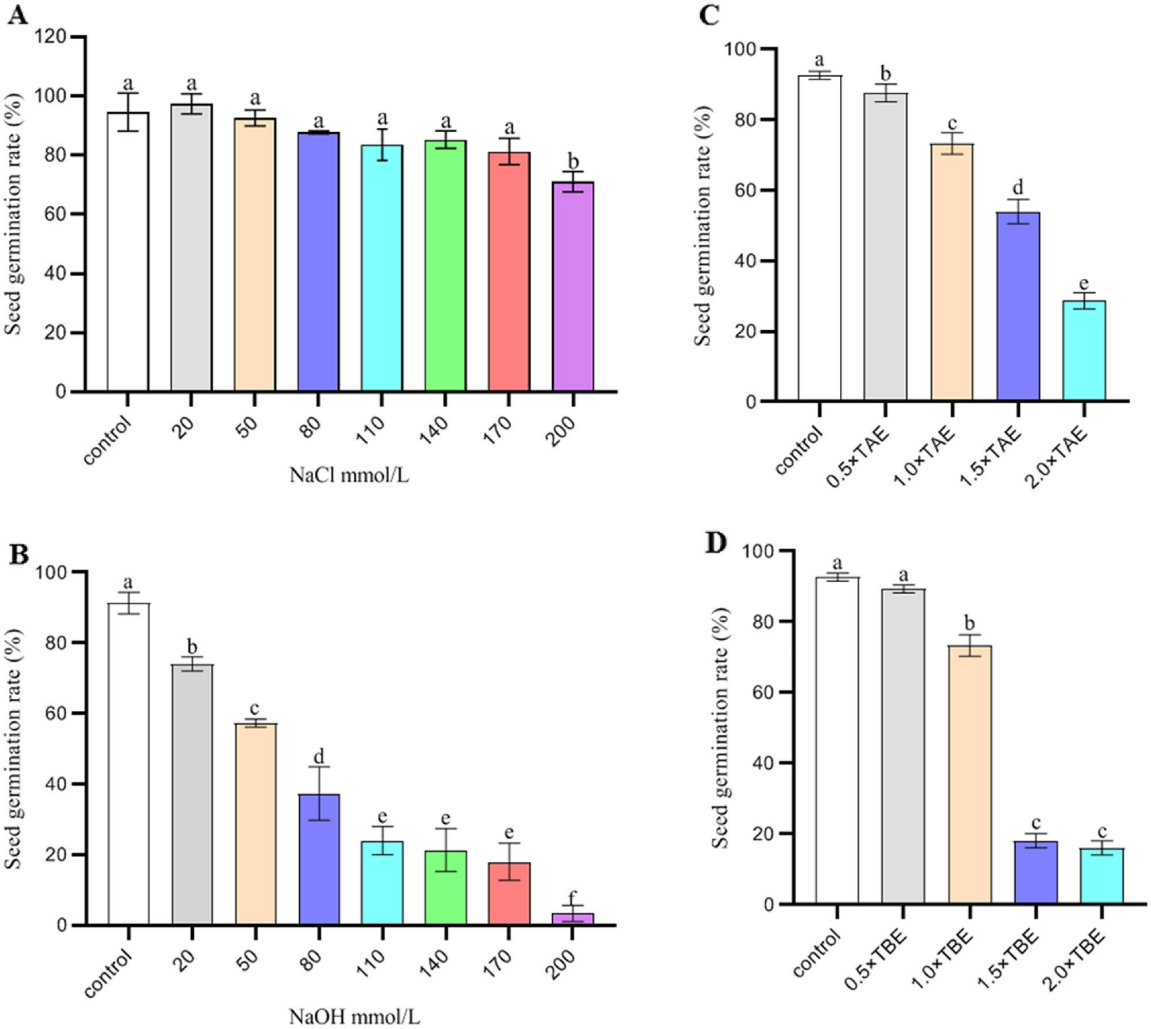
Germination rate of rice seeds at different solution concentrations. (**A**) NaCl solution; (**B**) NaOH solution; (**C**) TAE buffer; and (**D**) TBE buffer. The error bars stand for standard deviations. Control: sterile water, different letters in each column indicate significant differences between different treatments (*p* < 0.05; Duncan’s test).

Four buffer solutions, NaCl solution, NaOH solution, TAE buffer, and TBE buffer, were used to explore their effects on the germination of rice seeds, sterile water was used as the negative control. The results showed that the 20 mM NaCl solution was the most effective and accelerated the germination of rice seeds, followed by the NaOH solution, the germination rate (GR) showed a sharp downward trend with increasing concentrations of TAE and TBE buffers. It is worth noting that the effect of 20 mM NaCl solution on the germination rate of rice seeds was more pronounced than that of sterile water (Fig. 1). Therefore, 20 mM NaCl solution was selected as the buffer for DCEB.

**Figure 2:**
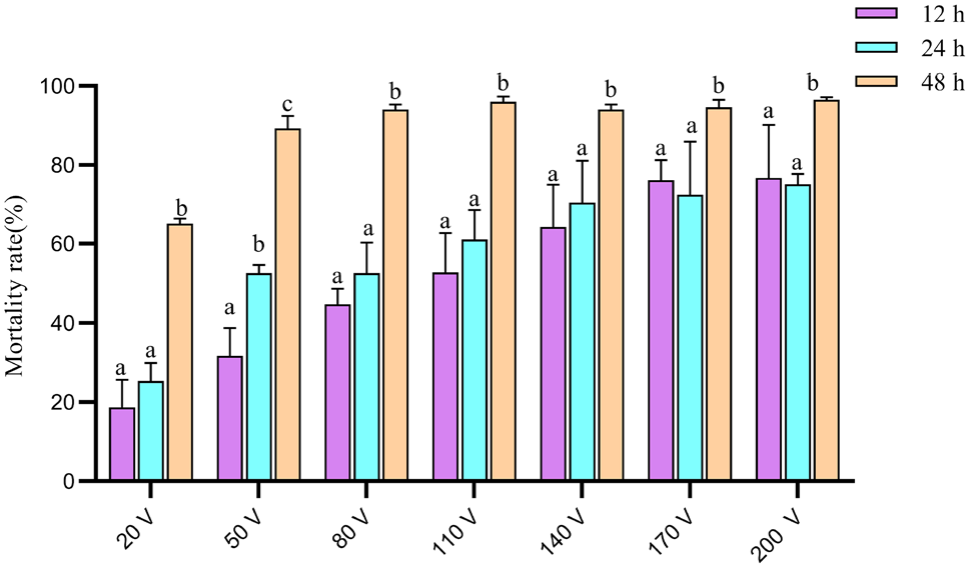
Mortality rate of germinated seeds after treating with DC electrophoresis bath under different voltage and processing times. The error bars stand for standard deviations, and the different letters on the top of the error bars mean significant difference at 5% level.

Seven voltages (20 V, 50 V, 80 V, 110 V, 140 V, 170 V and 200 V) and three treatment times (12 h, 24 h and 48 h) were used for a total of 21 treatment combinations, and 20 mM NaCl solution was used as the electrophoresis buffer. The results showed that the mortality of rice seeds followed an upward trend with increasing time and voltage, the increasing trend of mortality becomes flat as the voltage increases to 140 V, when the treatment time extend to 48 h, the mortality rate was all arrived 85% except 20 V, suggesting that the effect of voltage on mortality becomes insignificant when the time extend to 48 h. When treated under 110 v for 12 h, the mortality rate was 52.67%, when treated under 110 v for 50 V or 80 V, the mortality rates reached 52.5%, which were close to the LD50 (Fig. 2, Table 1).

### Effect of γ-irradiation and DC electrophoresis bath mutagenesis on rice seed vigor

**Figure 3:**
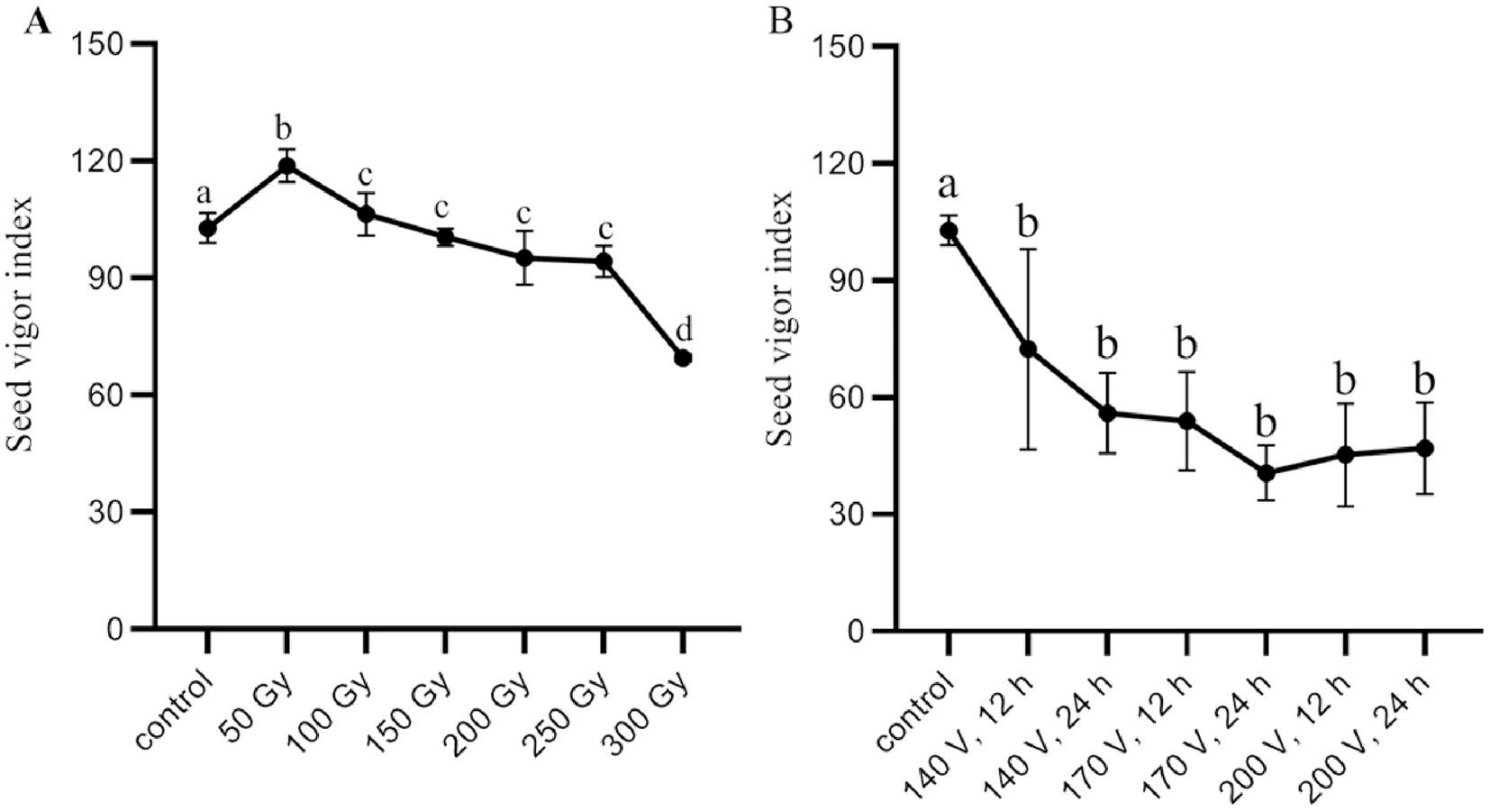
Seed vigor index after treated with (**A**) γ-irradiation; (**B**) DC electrophoresis bath. The error bars stand for standard deviations and the black dots (●) for mean values, different letters on the top of the error bars mean significant difference at 5% level (n = 3).

Finally, we selected six DCEB treatments with the three highest voltages (140 V, 170 V and 200 V), and six doses of γ-irradiation (50 Gy, 100 Gy, 150 Gy, 200 Gy, 250 Gy, and 300 Gy), as well as CK, consisting of thirteen treatments, were obtained to analyze seed vigor (Table 2). Compared with the control, γ-irradiation had a positive effect on rice seed vigor at 50 Gy and 100 Gy and a negative effect at other doses. The rice seed vigor index (VI) showed a trend of increasing and then decreasing with different doses of γ-irradiation (Fig. 3, Table 2). There were extremely significant differences between the positive and negative effects of the 50 Gy and 300 Gy doses, respectively (Table 3). This result indicates that when the dose of γ-irradiation reached 100 Gy, it had a positive effect on the seed vigor, and beyond this threshold, the seed vigor decreased. In contrast, DCEB mutagenesis had a negative effect on rice seed vigor under all six-level voltages treatments, the GR, GI and VI decreasing with increasing electrophoresis time and voltage (Fig. 2, Table 1). This result indicates that the both methods have effects on seed viability, and the DCEB mutagenesis has more negative significant effects on rice seed vigor than that γ-irradiation.

**Table 1:** The mortality rate (%) of germinated seeds after treating with DC electrophoresis bath under different voltage and processing times.

Treatments*	12 h	24 h	48 h
20 V	18.67 ± 5.73 a	25.33 ± 3.77 a	64.00 ± 1.00 b
50 V	31.67 ± 5.79 a	52.50 ± 1.50 b	91.70 ± 2.25 c
80 V	44.80 ± 3.11 a	52.50 ± 5.50 b	95.00 ± 1.00 b
110 V	52.67 ± 8.22 a	61.00 ± 6.16 a	95.70 ± 1.00 b
140 V	64.17 ± 8.78 a	70.33 ± 8.81 a	93.30 ± 1.00 b
170 V	76.00 ± 4.32 a	72.33 ± 11.12 a	93.30 ± 1.35 b
200 V	76.67 ± 11.03 a	75.00 ± 2.00 a	97.00 ± 0.50 b

*The different letters mean significant difference at 5% level.

**Table 2:** Seed vigor index after treated with γ-irradiation and DC electrophoresis bath.

Treatments	Repeat 1	Repeat 2	Repeat 3	Average*
CK	98.53	104.20	105.75	102.83 ± 3.10 a
DC electrophoresis bath				
140 V, 12 h	90.91	83.21	43.04	72.38 ± 20.99 b
140 V, 24 h	67.06	46.56	54.36	55.99 ± 9.45 b
170 V, 12 h	55.55	40.60	65.71	53.95 ± 10.31 b
170 V, 24 h	34.99	38.25	48.60	40.62 ± 5.80 b
200 V, 12 h	60.47	38.98	36.48	45.31 ± 10.77 b
200 V, 24 h	51.80	55.47	33.54	46.94 ± 9.59 b
γ-irradiation				
50 Gy	120.66	114.02	121.87	118.85 ± 3.45 b
100 Gy	100.25	110.36	108.55	106.39 ± 4.40 c
150 Gy	101.97	101.63	97.96	100.52 ± 1.82 c
200 Gy	94.54	102.36	88.60	95.17 ± 5.63 c
250 Gy	90.25	94.33	98.24	94.27 ± 3.26 c
300 Gy	68.47	70.39	69.80	69.59 ± 0.80 d

*The different letters mean significant difference at 5% level.

### Effect of γ-irradiation and DCEB mutagenesis on agronomic traits

We investigated the agronomic traits of M_1_ generations induced by DCEBs and γ-irradiation, respective. In Table 4, the number of productive tillers of plants treated with γ-irradiation were significantly higher than that of control plants, while plant height and filled grain percentage were lower in treated plants than the control; no significant differences were found for other agronomic traits, such as thousand-grain weight and flag leaf length and width. In particular, the 250 Gy and 300 Gy doses produced more productive tillers and lower filled grain percentages than the control. There was almost no significant effect of DCEB treatment on the contemporary agronomic traits compared to the control (Table 4).

**Table 3:** Effects of γ-irradiation and DC electrophoresis bath mutagenesis on seed viability across the different treatments.

Treatments	Germination rate (%)	Relative effect	Germination potential	Relative effect	Germination index	Relative effect	Bud length (cm)	Relative effect	Vigor index	Relative effect
WT	88.67 ± 1.33	0.00	76.00 ± 1.15	0.00	22.07 ± 0.47	0.00	4.65 ± 0.34	0.00	102.83 ± 2.19	0.00
50 Gy	86.67 ± 2.40	− 2.26	80.00 ± 1.76	5.26	24.50 ± 0.508	11.01	4.85 ± 0.19	4.30	118.85 ± 2.43**	15.57
100 Gy	90.67 ± 2.91*	4.62	79.33 ± 2.91	4.38	22.90 ± 0.66	3.76	4.64 ± 0.16	− 0.22	106.39 ± 3.11	3.46
150 Gy	86.00 ± 3.06	− 0.77	76.67 ± 0.67	0.88	22.56 ± 0.26	2.22	4.89 ± 0.15	5.16	100.52 ± 1.28	− 2.25
200 Gy	86.00 ± 5.29	− 0.77	73.33 ± 1.76	− 3.51	20.98 ± 0.88	− 4.94	4.53 ± 0.16	− 2.58	95.17 ± 3.98*	− 7.45
250 Gy	86.00 ± 1.15	− 0.77	78.67 ± 0.67	3.51	21.69 ± 0.53	− 1.72	4.34 ± 0.21	− 6.67	94.27 ± 2.31*	− 8.32
300 Gy	86.67 ± 0.67	0.00	76.67 ± 1.76	0.88	21,11 ± 0.17	− 4.35	3.29 ± 0.14**	− 29.25	69.58 ± 0.58***	− 32.33
140 V, 12 h	41.67 ± 9.38***	51.92	31.33 ± 7.68***	58.78	16.92 ± 3.47	− 23.33	4.27 ± 0.14	− 8.17	72.38 ± 14.84**	− 26.56
140 V, 24 h	32.00 ± 5.13***	63.08	21.33 ± 3.38***	71.93	12.78 ± 1.36**	− 42.09	4.38 ± 0.16	− 5.81	55.99 ± 5.97***	− 43.19
170 V, 12 h	28.00 ± 3.05***	67.69	25.66 ± 2.85***	66.24	13.06 ± 1.76**	− 40.82	4.13 ± 0.12*	− 11.18	53.95 ± 7.29***	− 45.26
170 V, 24 h	25.00 ± 1.00***	71.15	14.00 ± 3.06***	81.58	9.69 ± 0.97***	− 56.09	4.19 ± 0.18	− 9.89	40.61 ± 4.10***	− 58.80
200 V, 12 h	27.00 ± 5.03***	68.85	23.66 ± 3.48***	68.87	11.42 ± 1.92**	− 48.26	3.97 ± 0.04*	− 14.62	45.31 ± 7.61***	− 54.03
200 V, 24 h	22.00 ± 3.21***	74.62	20.00 ± 3.21***	73.68	11.28 ± 1.62**	− 48.89	4.16 ± 0.15	− 10.54	46.93 ± 6.78***	− 52.38

Data are expressed as mean ± S.E. *p < 0.05, **p < 0.01, and ***p < 0.001.

### Physiological damage caused by γ-irradiation and DCEB mutagenesis

The combination of seed vigor performance and agronomic traits in the M_1_ generation suggested that γ-irradiation had a significant effect on the agronomic traits; while the DCEB had a significant effect on seed vigor index. When estimating the physiological damage caused by both mutagenesis methods, the physiological damages (germination rate, bud length, panicle weight and filled grain percentage) increased with increasing doses of γ-irradiation and showed a linear correlation. Moreover, the physiological damages caused by DCEB increased with higher voltage and a longer electrophoresis time.

**Table 4:** Effects of γ-irradiation and DC electrophoresis bath mutagenesis on major agronomic traits.

Treatments	Plant height (cm)	Flag leaf length (cm)	Flag leaf width (cm)	Panicle weight (g)	No. of productive tillers	panicle length (cm)	Filled grain percentage (%)	Thousand-grain weight (g)
WT	80.33 ± 0.77	23.39 ± 1.34	1.70 ± 0.22	23.90 ± 0.97	8.3 ± 0.50	15.20 ± 0.26	0.89 ± 0.08	25.21 ± 0.64
50 Gy	78.90 ± 0.81	23.36 ± 0.93	1.81 ± 0.11	29.91 ± 1.59*	11.9 ± 0.55*	15.10 ± 0.22	0.84 ± 0.02	26.83 ± 0.47
100 Gy	77.62 ± 1.43	24.14 ± 0.58	1.81 ± 0.06	26.22 ± 2.06	11.3 ± 0.75*	15.00 ± 0.21	0.75 ± 0.05*	27.16 ± 0.40
150 Gy	73.40 ± 0.78**	21.60 ± 1.16	1.71 ± 0.12	21.73 ± 1.91	11.5 ± 1.17*	14.91 ± 0.31	0.65 ± 0.03**	26.85 ± 0.22
200 Gy	76.74 ± 0.93*	23.89 ± 0.96	1.73 ± 0.09	24.00 ± 1.76	11.2 ± 0.68*	15.62 ± 0.21	0.68 ± 0.06**	27.23 ± 0.42
250 Gy	76.96 ± 1.38*	20.60 ± 1.61	1.70 ± 1.24	9.87 ± 1.08**	15.4 ± 1.54**	13.70 ± 0.36**	0.13 ± 0.05***	24.56 ± 1.22
300 Gy	76.89 ± 0.54*	23.40 ± 1.73	1.62 ± 0.19	11.58 ± 1.79**	14.9 ± 1.46**	14.38 ± 0.42*	0.16 ± 0.06***	24.15 ± 0.52
140 V, 12 h	78.30 ± 1.72	25.30 ± 1.57	1.94 ± 1.51*	23.60 ± 1.60	8.6 ± 0.51	15.24 ± 0.29	0.82 ± 0.03	21.88 ± 0.45*
140 V, 24 h	81.26 ± 1.14	21.56 ± 3.49	1.82 ± 0.36	29.68 ± 1.93*	10.2 ± 0.37	16.20 ± 0.31*	0.90 ± 0.01	24.47 ± 0.39
170 V, 12 h	79.94 ± 1.60	29.28 ± 2.77*	1.58 ± 0.35	29.14 ± 1.48	10.0 ± 0.45	16.51 ± 0.28*	0.93 ± 0.01	25.65 ± 0.48
170 V, 24 h	79.43 ± 0.48	24.20 ± 0.78	1.73 ± 0.05	22.56 ± 6.10	9.6 ± 2.72	14.76 ± 0.51	0.93 ± 0.02	27.56 ± 6.35
200 V, 12 h	79.26 ± 1.76	21.18 ± 1.28	1.74 ± 0.05	23.24 ± 1.78	9.0 ± 0.55	15.83 ± 0.30	0.91 ± 0.01	23.62 ± 0.41
200 V, 24 h	78.02 ± 0.70	22.78 ± 1.08	1.72 ± 0.18	22.57 ± 2.91	10.4 ± 1.03	16.07 ± 0.16	0.92 ± 0.01	23.80 ± 0.53

Data are expressed as mean ± S.E. *p < 0.05, **p < 0.01, and ***p < 0.001.

### Genetic background analysis of mutants

**Figure 4:**
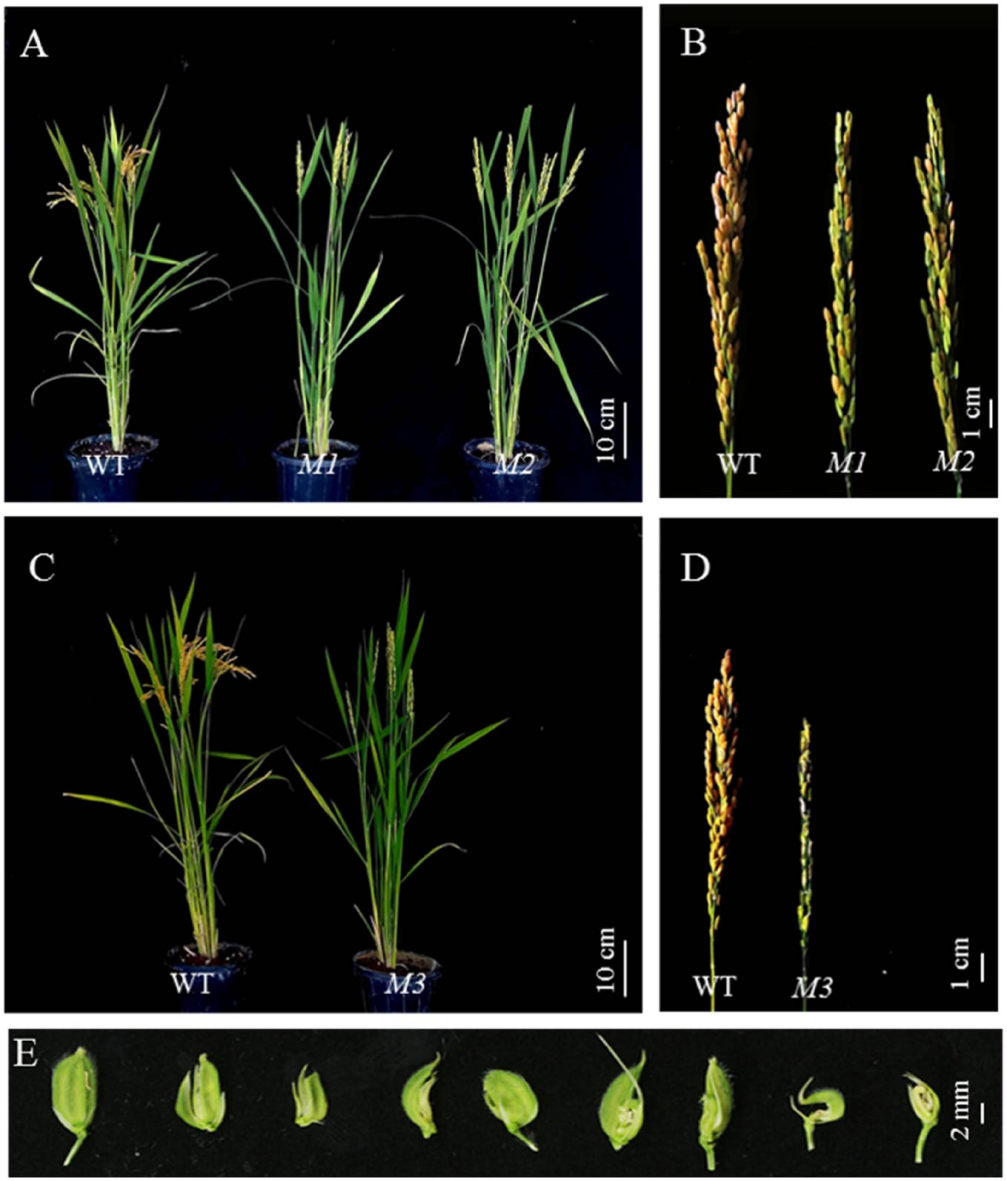
The Mutants of low filled grain percentage (*M1* and *M2*) and abnormal hull (*M3*). (**A**) The plant type of *M1* and *M2* (right two) compared with WT (left); (**B**) The panicle shape of *M1* and *M2* (right two) compared with WT (left); (**C**) The plant type of *M3* (right) compared with WT (left); (**D**) The panicle shape of *M3* (right) compared with WT (left); and (**E**) The deformed glumes of *M3*. WT, wild type, *M1*, *M2* and *M3* were obtained from M_2_ generations which were self-crossed from M_1_ generations treated by DCEB of140 V, 48 h, 200 V, 48 h and 170 V, 48 h, respectively.

**Figure 5:**
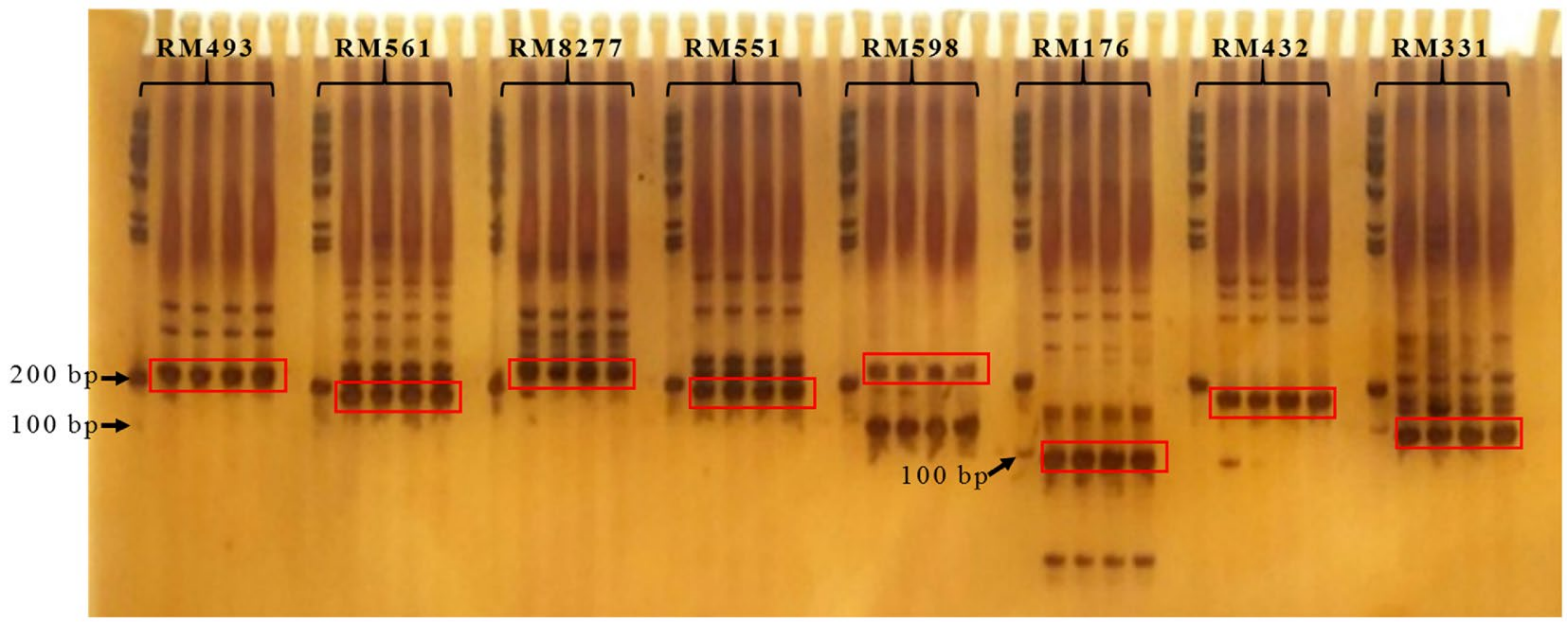
Parts of gel electropherograms profile of wild type and mutant lines. For each SSR maker, lanes from left to the right are: M, W, *M1* and *M2*. M: DNA ladder. M: DNA ladder 1000; W: wild type; *M1* and *M2*: low filled grain percentage mutants from 140 V, 48 h treatment and 2 from 140 V, 48 h treatment, respectively; *M3*: abnormal hull mutant from 110 V, 48 h treatment. The red boxes indicate the main amplified blots for each SSR, and the whole gel electropherograms profile and the original gels are presented in Supplementary Figure S2.

There were 116 seeds germinated with 102 healthy seedlings obtained from the DCEB treatment of 140 V, 48 h; 92 seeds germinated with 78 healthy seedlings and 72 seeds germinated with 60 healthy seedlings were obtained from the treatments of 170 V, 48 h and 200 V, 48 h, respectively. There was no apparent mutation from each M_1_ generations, however, from M_2_ generation (self-crossed from M_1_ generation)we screened two mutants (*M1,* from the treatment of 140 V, 48 h, and *M2,* from the treatment of 200 V, 48 h,) with low filled grain percentage and one mutant (*M3,* from the treatment of 170 V, 48 h) with abnormal hull. The fertile pollen percentage of *M1*, *M2*, *M3* and WT was about 36.5%, 44.1%, 56.7% and 97.6%, respectively, and the filled grain percentage was 6.3%, 13.4%, 26.8% and 93.2%, respectively (Fig. 4). The genetic analysis of the mutants according to the 48 SSR markers showed that all three mutants shared the wild type bands (Fig. 5). These results clearly indicated that all three mutants are true mutants.

### Evaluation of resequencing results

To further investigate the effects of DCEB mutagenesis at the genetic level, three experimental materials (WT, *M1* and *M2*) were subjected to WGR in this study. Approximately 37.43 Gbp of clean data was produced, with a Q20 of 97.03%, Q30 of 92.25% and GC of 42.43% (averaged from WT, *M1* and *M2*, respectively, Table 5). According to the results of WGR, the average sample-to-reference genome match was 98.70%, the average depth of coverage was 29X, and the genome coverage was 97.15%. The above results indicate that the sequencing data are reliable and can be used for SNP and InDel analysis.

**Table 5:** Sequencing data evaluation statistics.

No	Clean reads	Clean base	Q20 (%)	Q30 (%)	GC (%)
WT	40,058,683	11,999,309,468	97.27	92.72	42.42
*M1*	41,188,439	12,337,303,412	97.03	92.24	42.14
*M2*	43,714,624	13,093,555,906	96.79	91.80	42.74

**Figure 6:**
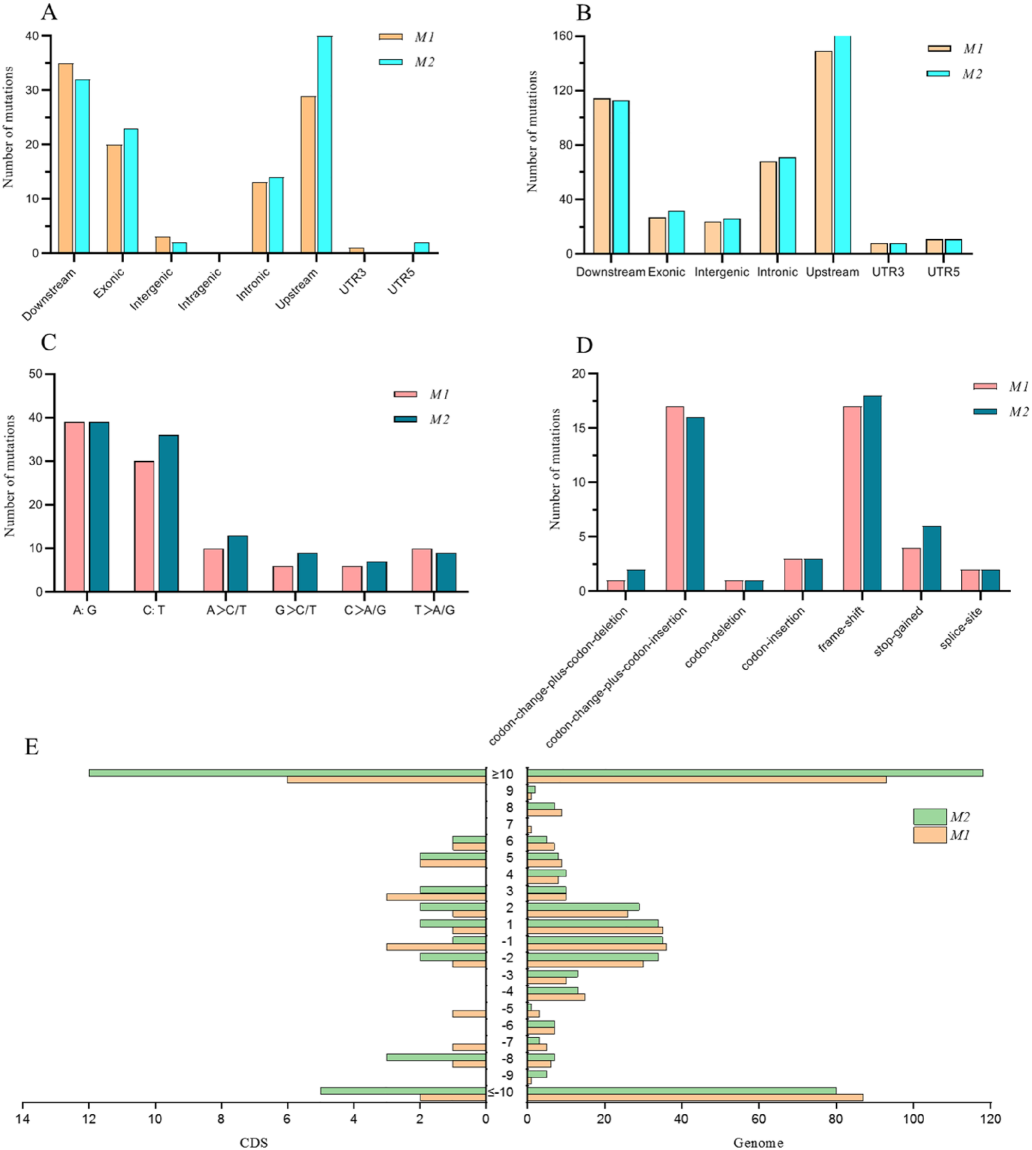
Mutations produced by DC electrophoresis bath mutagenesis. (**A**) SNPs variant types in the genome; (**B**) InDels variant types in the genome. (**C**) Mutation of different categories of SNPs induced by DC bath; (**D**) InDels variants in the genetic region; and (**E**) InDels length distribution in CDS and genome.

Mutations that were identical to those of the wild type were filtered out. After filtering, the *M1* mutant had a total of 101 SNPs and 402 InDels, and the *M2* mutant had a total of 113 SNPs and 424 InDels. The mutation frequencies of *M1* and *M2* were calculated to be 2.39 × 10^–6^ and 2.56 × 10^–6^ (Calculated according to reference^30^), respectively. The chromosome with the highest number of mutations was Chr7, followed by Chr9 and Chr12, and the chromosomes with the lowest number of mutations were Chr8 and Chr6 (the distribution of the SNPs and InDels on each chromosome is shown in Supplementary Figure S1). SNP variants was present at the highest levels in downstream regions, followed by upstream regions, exon regions and intergenic regions. Meanwhile, a large proportion of the InDel variants were found in downstream and upstream regions and only about 7% was present in exon regions (Fig. 6A,B). SNP variants occupied 20% of the mutation sites in the exon region, which indicated that the effect of DCEB mutagenesis on the function of rice genes is relatively large.

### SNP and InDel variants of the two mutants

In contrast to the wild type, among the two mutants *M1* and *M2*, SNP mutations included 78 A to G transition (36.4%), 66 C to T transition (30.8%); and 23 A > C/T transversion (10.7%), 15 G > C/T transversion (7.0%), 13 C > A/G transversion (6.1%), and 19 T > A/G transversion (8.9%). In both mutants, A: G transitions were the most abundant type of mutations detected by DC bath, followed by the C:T transitions. The most frequent types of transversions in *M1* mutants were A > C/T and T > A/G, and in *M2* mutants, the most frequent type of transversions was A > C/T (Fig. 6C).

In both mutants, InDels mutations in the coding region were analyzed, and frame-shift were the most common type of mutation, accounting for approximately 37% of the entire coding region (Fig. 6D). The longest insertion mutation in the coding region was a frame-shift mutation at 97 bp, and the longest deletion mutation in the coding region was a stop-gained mutation at 99 bp. The most common mutations are 1–2 bp insertion and deletion mutations (Fig. 6E).

## Discussion

### Definition of the optimal median lethal dose for DCEB

According to the definition of median lethal dose (LD50), the result should be 50 V and 80 V for 24 h and 140 V for 12 h for DCEB, under which the seed germinations were close to 50%, however we didn’t screen the significant phenotypically mutants in the M_2_ generation of these treatments; while *M1*, *M2*, *M3* and other types of suspected mutants appeared in 140 V, 200 V and 140 V for 48 h. The mutagenic effect on rice was further increased along with the increasing voltage and processing time in treatments above the LD50, so that we easily obtained significant phenotypically mutants. Therefore, LD50 is not necessarily suitable for DCEB mutagenesis, and a higher lethal dose (higher voltage or/and longer processing time) can be taken appropriately to be able to increase the probability of mutagenesis.

### Effects of γ-irradiation and DCEB on physiological damage

This study investigated the effect of two methods (γ-irradiation and DCEB) on the physiological damages to rice seeds. The results showed that at low doses, γ-irradiation had a positive effect on the germination potential and germination index (GI), while high doses of γ-irradiation significantly reduced the shoot length and vigor index, which is consistent with the results of previous studies^31–33^. However, rice seeds treated with γ-irradiation in this study all had significantly higher panicle numbers and lower filled grain percentage than controls, especially after a dose of 250 Gy. These results may be due to the high doses of γ-irradiation causing pollen sterility^34,35^. In contrast, the rice seeds treated with the DCEB method all had extremely significantly lower values for rice seed viability, and the performance of the contemporary agronomic trait was essentially indistinguishable. The result suggests that γ-irradiation mainly affects contemporary agronomic traits, whereas the DCEB mainly affects rice seed vigor.

### The effect of DCEB mutagenesis on gene function

In present study, SNPs were found to be the most frequent mutation type, with C: T > A: G being the most frequent type of transitions, which is consistent with the results of other common mutagens. Transitions occurred more frequently than transversions, with an observed transition/transversion ratio of 2.0. For InDels, 1–2 bps insertions or deletions were detected most frequently, and the longest insertion in the coding region was a 97 bps frameshift mutation, while the longest deletion was a 100 bps deletion with a stop codon gain mutation. The DCEB method produced a large proportion of mutations detected in the exon regions of genes, particularly for SNP variants, with 20 (19.8%) mutations on *M1* and 23 (20.4%) on *M2* were screened.

### The advantages of DCEB

Compared with traditional irradiation mutagenesis methods^36^, DCEB only needs electrophoresis instrument to mutagenize, which has the advantages of low requirements for instruments and equipment, simple, convenient and flexible operation, safe experimental environment, low experimental cost, etc. According to the reported studies, the number of mutations in exon regions is less than 10% of the genome mutations caused by γ-radiation^37,38^, DCEB can cause about 20% exon mutation, this result suggests that the effect of DCEB mutagenesis on gene function in rice is relatively large, especially the mutagenic effect is apparent at a treatment time of 48 h. It provides a new method for future mutagenesis breeding in rice and even in other crops.

## Conclusions

The present study provides a new mutagenesis method by using DCEB. We identified three mutants (*M*1, *M*2 and *M*3) from M_2_ generation and did the WGR for *M*1 and *M*2. *M*1 had a total of 101 SNPs and 402 InDels, and the *M*2 had a total of 113 SNPs and 424 InDels, indicating that insertional or deletion mutations were the main type of mutations as induced by DCEB. The mutation frequencies were calculated at 2.39 × 10^–6^ and 2.56 × 10^–6^ for *M*1 and *M*2, respectively. Meanwhile, the exon region of mutant genes occupied a large proportion, especially for SNP variants, which occupied about 20% of the mutation sites in the exon region. The DCEB may provide a simple, convenient, safe, flexible, and low-cost method for future mutagenesis breeding in rice and even in other crops.

## Materials and methods

### Experimental materials

In this study, XiangGeng 365, a cultivated variety rice (*Oryza sativa* L. ssp. *japonica*) was used as experimental material. We obtained the seeds from the National Engineering Research Center of Plant Space Breeding, South China Agricultural University (Guangzhou, China).

### DCEB treatment of seeds

**Figure 7:**
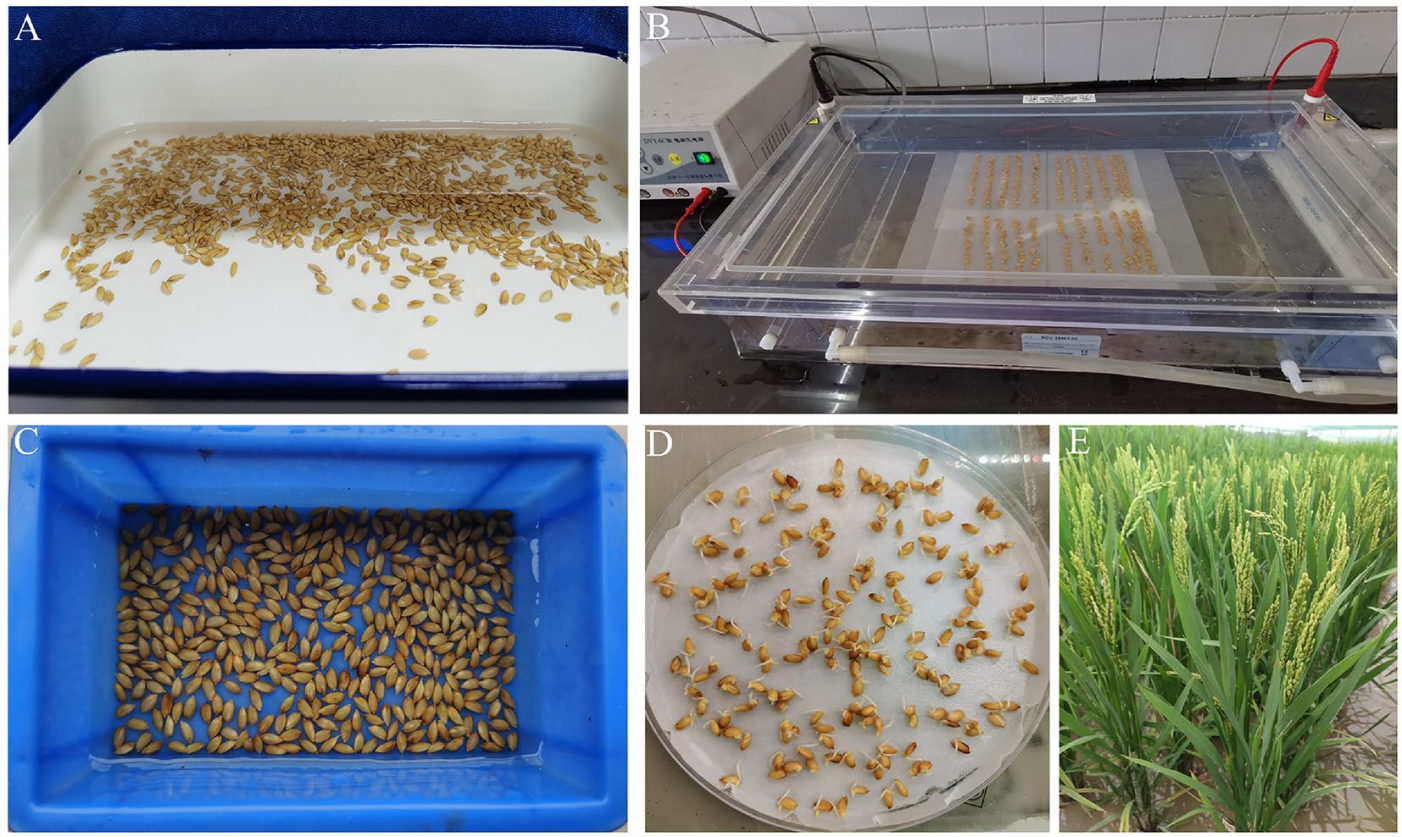
Protocol for the DC electrophoresis bath. (**A**) Soak the rice seeds in clean water for 24 h and disinfected with 3–6% sodium hypochlorite for 30 min; (**B**) the soaked and disinfected seeds were placed in an electrophoresis tank lined with filter paper and buffer, and then start the DC electrophoresis for a certain period of time; (**C**) After electrophoresis, the buffer residue was removed by washing with water; (**D**) Seeds were placed on petri dishes lined with filter paper for germination, and (**E**) Seedlings from these germinated seeds were transplanted to the field.

Plump seeds of XiangGeng 365 were soaked in water for 24 h and disinfected with 3–6% sodium hypochlorite for 30 min. For each DCEB treatment, 400 seeds were selected and placed in an electrophoresis tank lined with filter paper and buffered for various processing time, after which the buffer residue was removed by washing with water. Then, the seeds were placed on petri dishes lined with filter paper for germination, and physiological indexes such as the GR and GI were calculated. Plants, named as M_1_ generation that germinated normally were transplanted to the field (Fig. 7), harvested when mature and examined for agronomic traits including plant height, filled grain percentage and thousand grain weight; and around one thousand plants of each M_2_ generation (obtained from the self-crossed M_1_ plants) were transplanted in the field for screening of mutations.

### γ-irradiation of seeds

In February 2021, rice seeds were irradiated in a γ-irradiator provided by Guangzhou Huada Biological Company. Each well of a 450-well plate filled with 1000 seeds were exposed to different doses of γ-irradiation ranging from 50 to 300 Gy with an interval of 50 Gy.

### The mutagenic effects induced by γ-irradiation and DCEB on M_1_ generation

The VI including GR, GI, bud length, and agronomic traits of M_1_ generations including plant height, flag leaf length and width, panicle weight, panicle length, number of productive tillers, filled grain percentage and thousand-grain weight were compared between the two methods. The mutagenic effects of DCEB and γ-irradiation on the physiological damage to seeds were estimated by quantifying four parameters on M_1_ plants: GR, bud length, panicle weight and filled grain percentage. The formula for calculating each physiological index is as follows^39^:

1 $${\text{GR}}(\% ) = \frac{{{\text{means}}\;{\text{of}}\;{\text{germinated}}\;{\text{seeds}}}}{{{\text{means}}\;{\text{of}}\;{\text{total}}\;{\text{seeds}}}} \times 100$$

2 $$\mathrm{GI}=\sum (\text{Gt/Dt})$$

3 $${\text{VI}} = {\text{S}} \times {\text{GI}}$$

Gt is the number of sprouts per day during the germination terminal period; Dt is the corresponding number of sprouting days; and S is the length of the seedling at germination time t.

### Screening of mutants and analysis of its genetic background

In March 2021, rice seeds were treated by DCEB method with seven different voltages (20 V, 50 V, 80 V, 110 V, 140 V, 170 V and 200 V) for 12 h, 24 h and 48 h for each voltage, respectively. A total of 21 treatments, including a control (treating with sterile water and no voltage), were performed. The suspected mutants were obtained from the M_2_ generations, and were further analyzed for genetic background compared with its wild type (WT) to ascertain whether they are true mutations by using 48 SSR markers provided by the protocol for identification of rice varieties issued by China in 2014, NY/T 1433–2014^40^.

### Whole-genome resequencing of mutants

The constructed DNA libraries were sequenced by the Illumina sequence platform at Biomarker Technologies Co., Ltd. (Beijing, China).

Make quality evaluation and filtering for the raw reads (Paired ends) obtained from sequencing to get Clean Reads, which can be used for subsequent bioinformatics. Align Clean Reads with reference genome, perform variation detection and annotation for SNP, InDel of aligned result to perform DNA-level DEGs mining and DEGs function annotation.

Raw sequenced reads or raw reads were obtained from sequencing, which also have low quality reads with adaptors. To ensure bioinformatics quality, raw reads were filtered to get clean reads for subsequent bioinformatics. The raw reads (double-ended sequences) from the sequences were quality assessed and filtered to obtain clean reads, which were compared with the reference genome sequence (MSU 7.0) for subsequent bioinformatics analysis^30^. The main steps of data filtering are as follows: (1) remove the reads with adapter; (2) filter reads with N content over 10%; (3) remove reads whose base value (with quality value less than 10) is more than 50%.

### Ethics approval

Experimental research and field studies on plants, including the collection of plant material, comply with relevant institutional, national, and international guidelines and legislation.

## Supplementary Information

Supplementary Information.

## Data Availability

All of the material is owned by the authors and/or no permissions are required. The data used to support the findings of this study are available from the corresponding author upon request.
