# Supplementary material for: A new method for mutation inducing in rice by using DC electrophoresis bath and its mutagenic effects
Source: Sci Rep. 2023 Apr 25;13:6707. doi: 10.1038/s41598-023-33742-7 (PMC10126576; doi:10.1038/s41598-023-33742-7)

**Supplementary Information**

**A new method for mutation inducing in rice by using DC electrophoresis bath and its mutagenic effects**

**Minmin Zou^✝^, Sun Tong^✝^, Ting Zou, Xinyi Wang, Linxuan Wu, Jiafeng Wang, Tao Guo, Wuming Xiao, Hui Wang, Ming Huang^*^**

Guangdong Provincial Key Laboratory of Plant Molecular Breeding, South China Agricultural University, Guangzhou, 510642, P.R. China

**Supplementary Figure Legend**

**Supplementary Figure S1**

The distribution of the SNPs and InDels on each chromosome. (**A**) Distribution of SNPs of *M1*; (**B**) Distribution of SNPs of *M2*; (**C**) Distribution of InDels of *M1*; and (**D**) Distribution of InDels of *M2*.

**Supplementary Figure S2**

Gel electropherograms profile of wild type and mutant lines. SSR markers for each gel from left to the right are: (**A**) RM583, RM71, RM85, RM471, RM274, RM190, RM336, RM72; (**B**) RM219, RM311, RM209, RM19; (**C**) RM1195, RM208, RM232, RM119, RM267, RM253; (**D**) RM481, RM339, RM278, RM258, RM224, RM17; (**E**) RM493, RM561, RM8277, RM551, RM598, RM176, RM432, RM331; (**F**) OSR28, RM590, RM21, RM3331; (**G**) RM443, RM490, RM424, RM423, RM571, RM231; (**H**) RM567, RM289, RM543, RM316, RM332, RM7102. For each SSR marker (**A-F**) lanes from left to the right are: M, W, *M1* and *M2* respectively.(**G**, **H)** the first lane is M, the rest lanes from left to the right are W, *M1* and *M2*respectively.M: DNA ladder1000; W: wild type; *M1* and *M2*: low filled grain percentage mutants from 140 V, 48 h treatment and 200 V, 48 h treatment, respectively; *M3*: abnormal hull mutant from 170 V, 48 h treatment; red boxes indicate the main amplified blots for each SSR marker.

Supplementary Figure S1


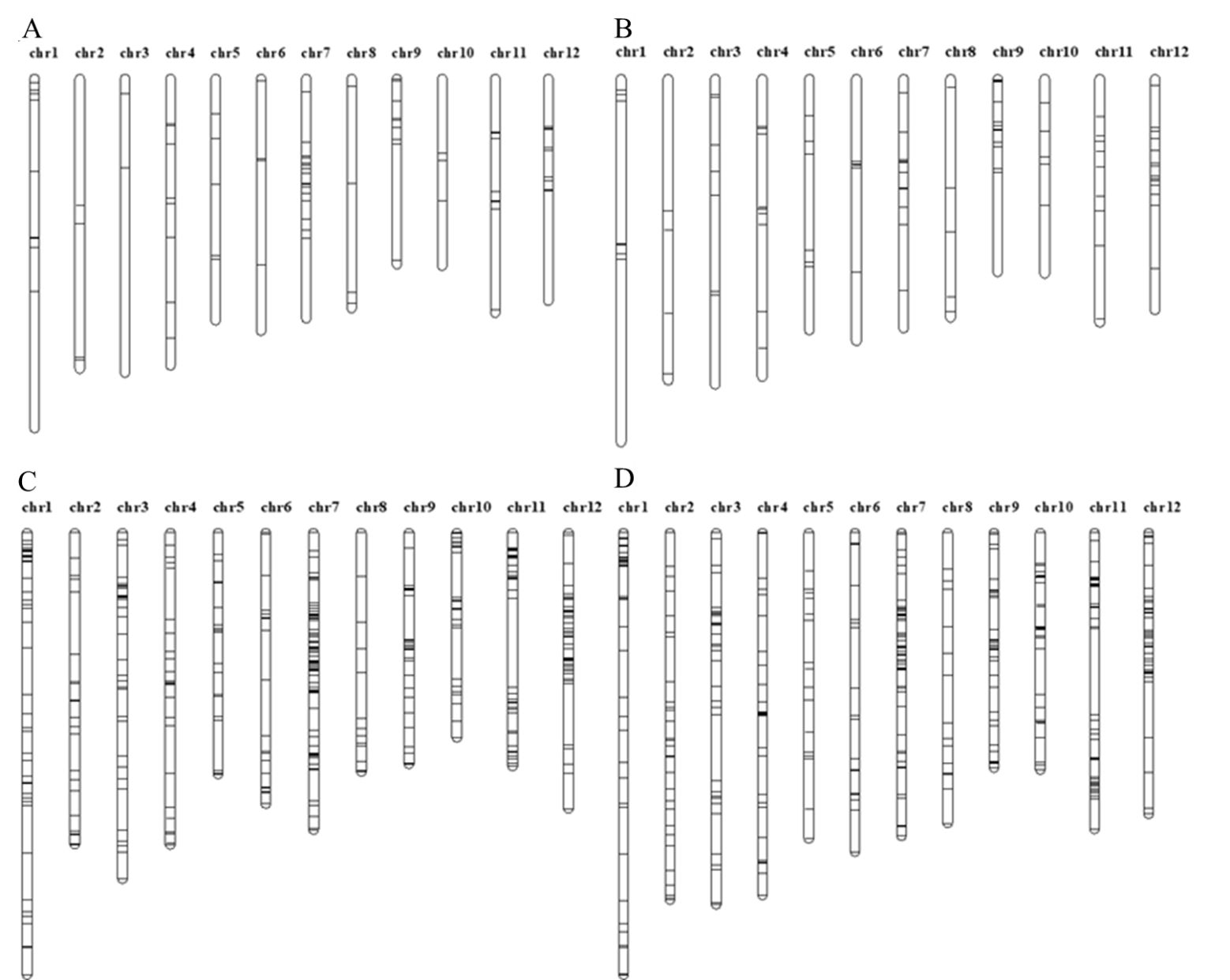


Supplementary Figure S2


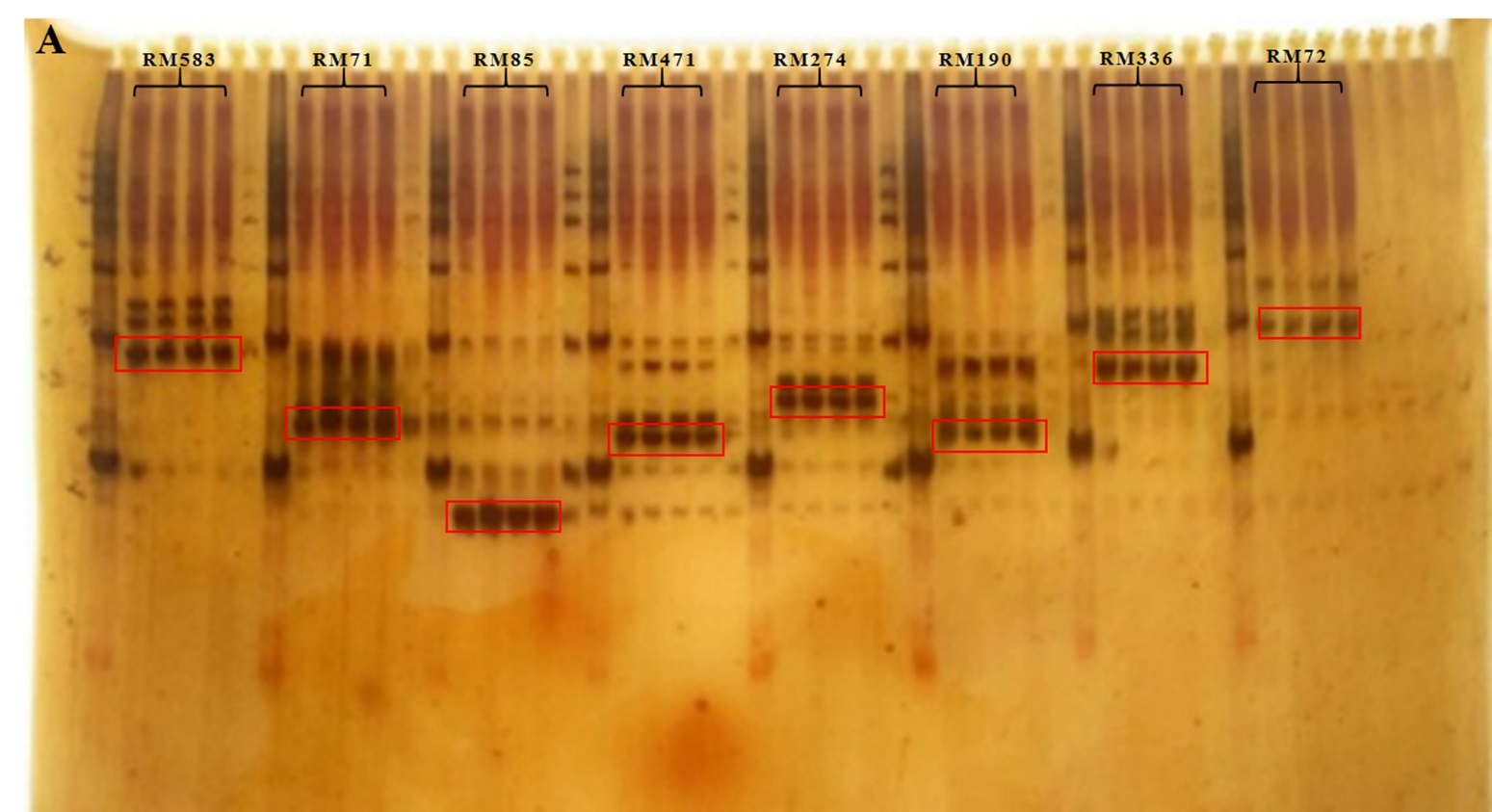


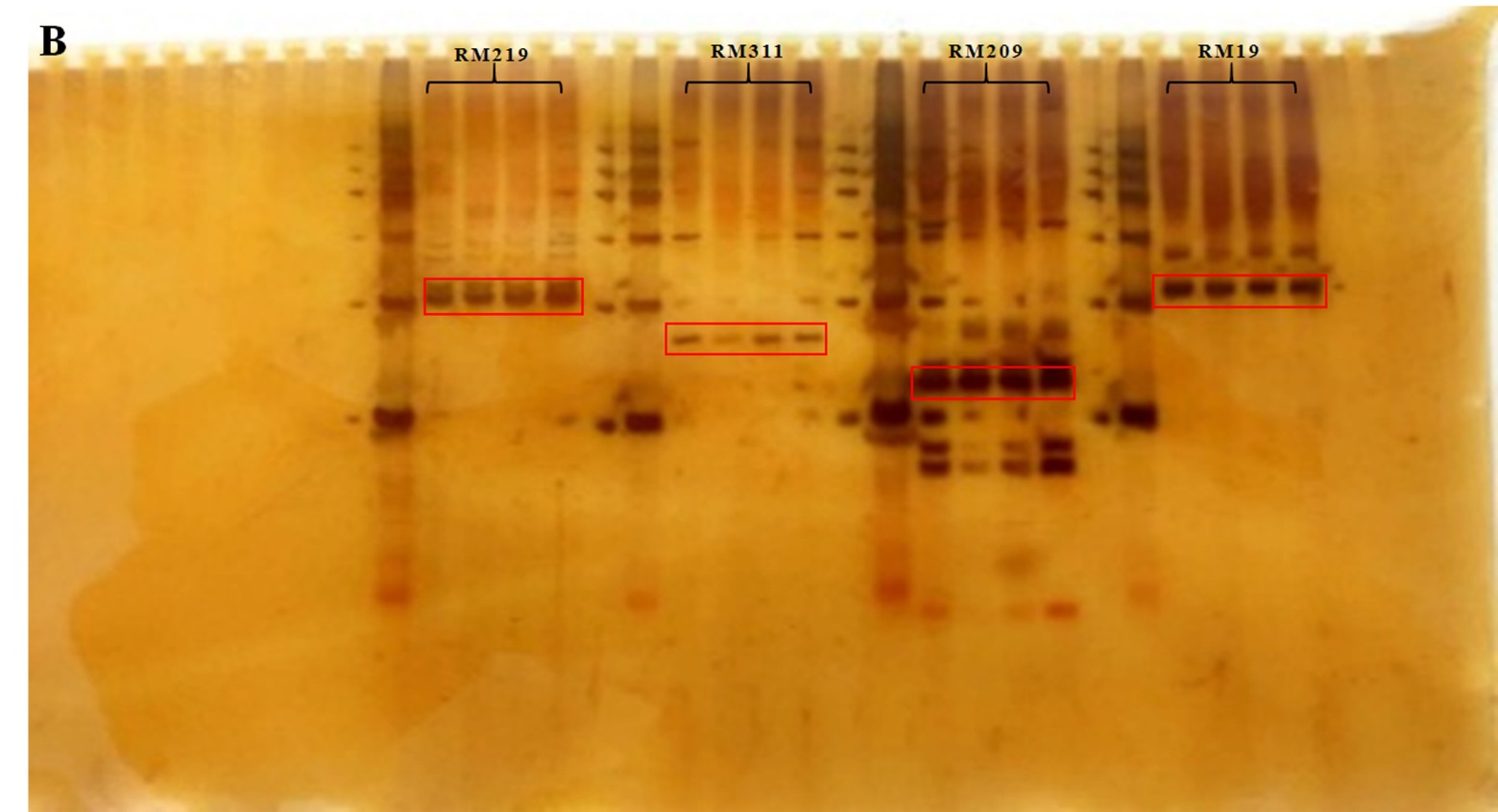


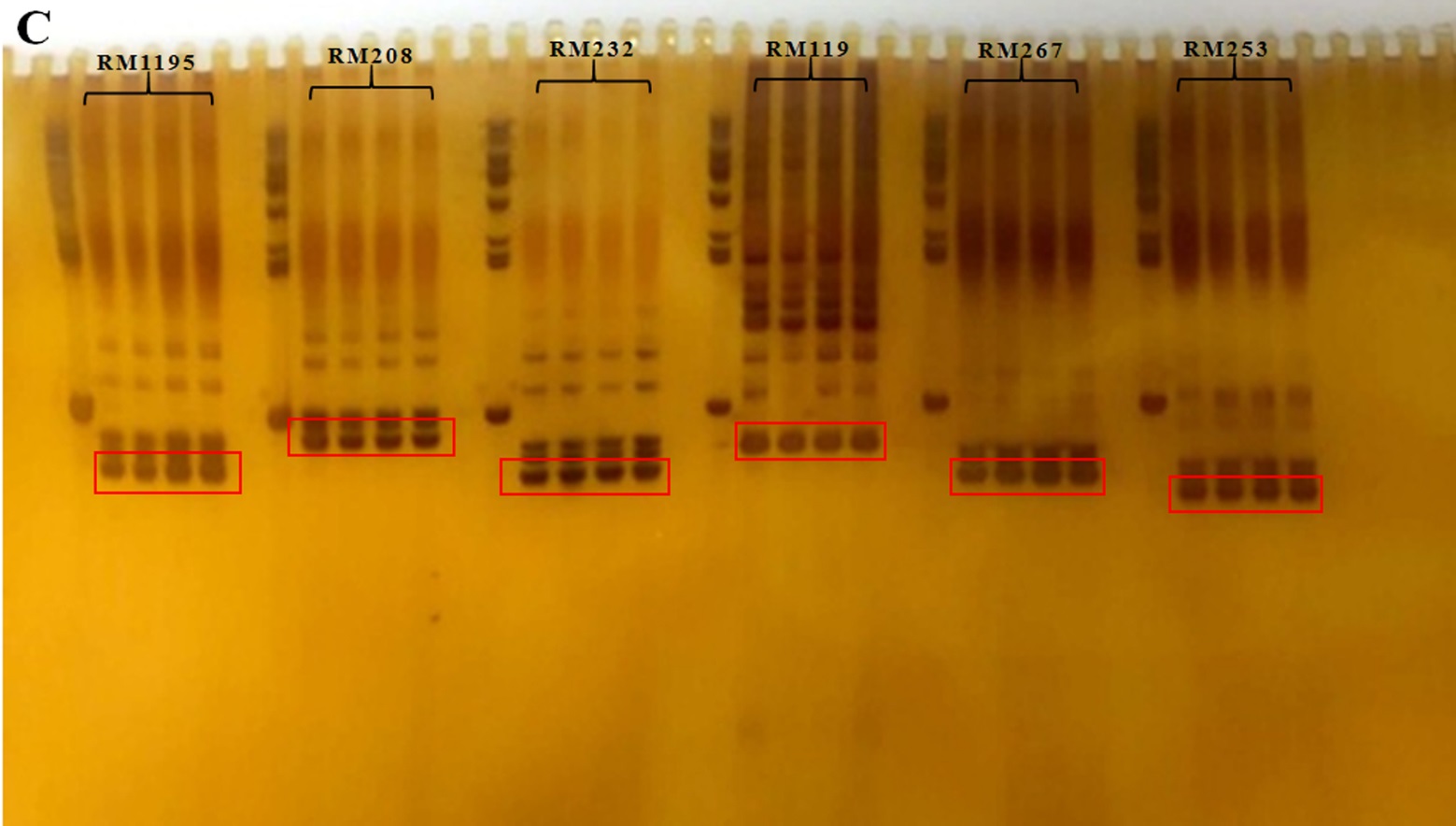


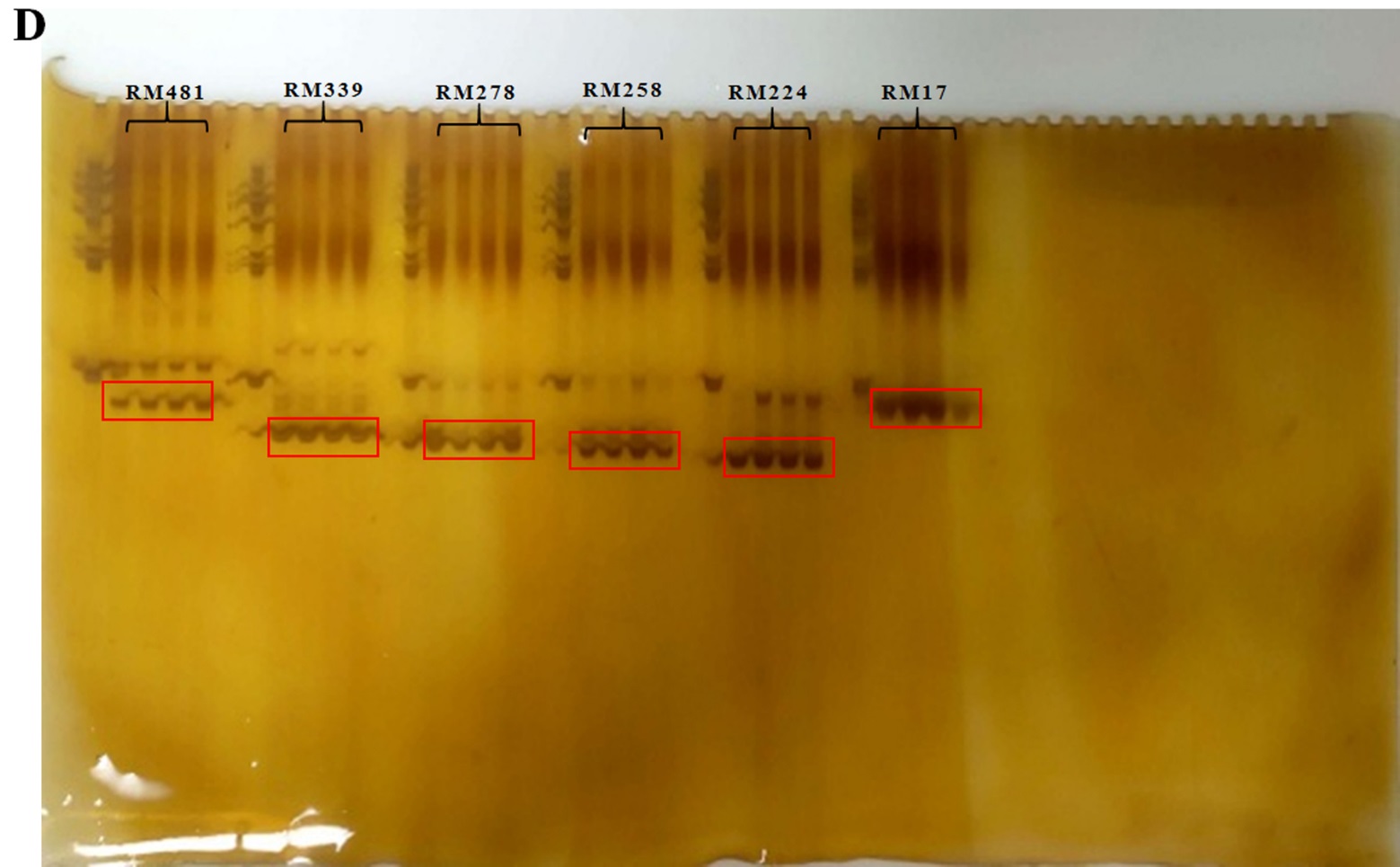


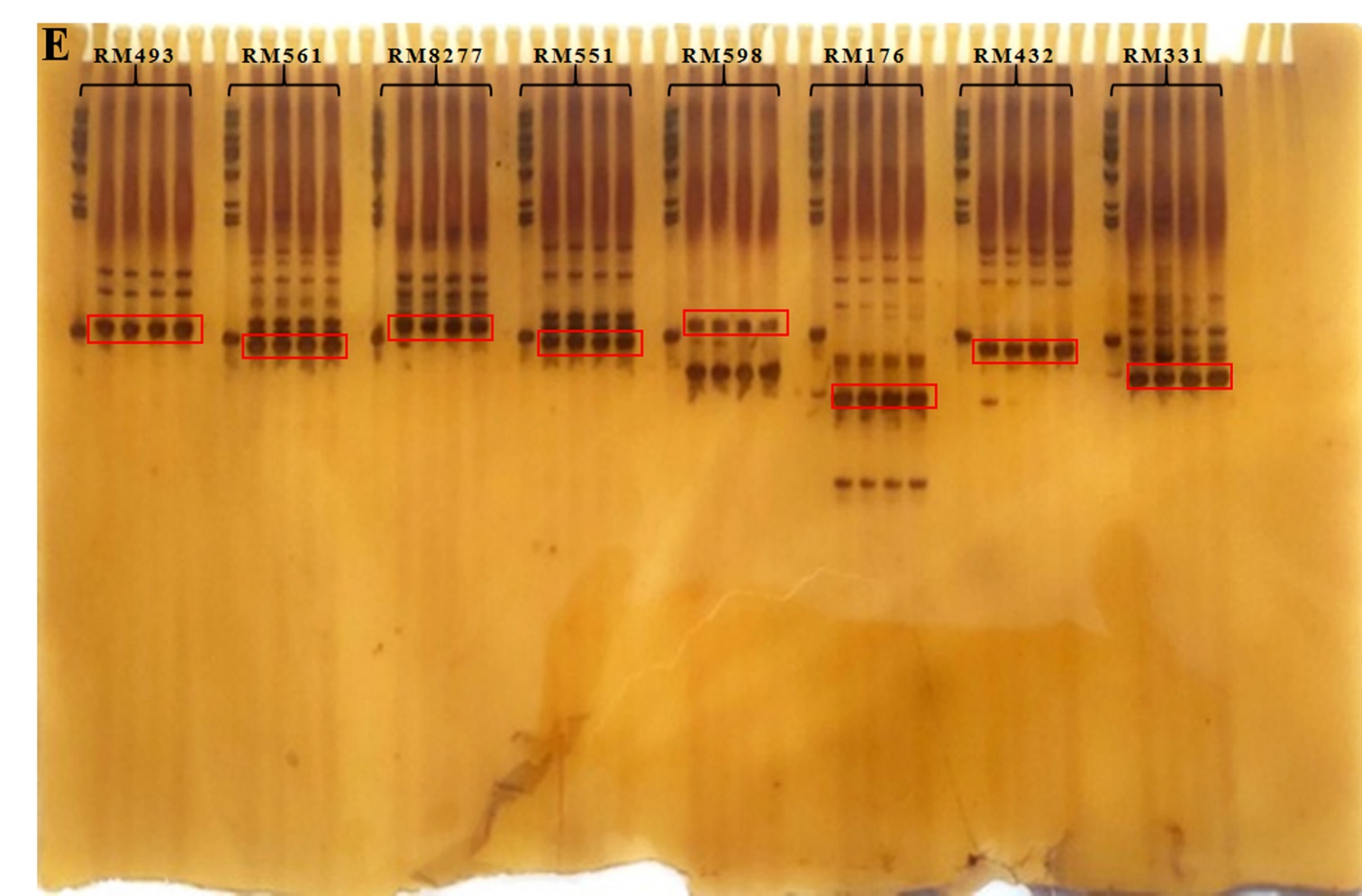

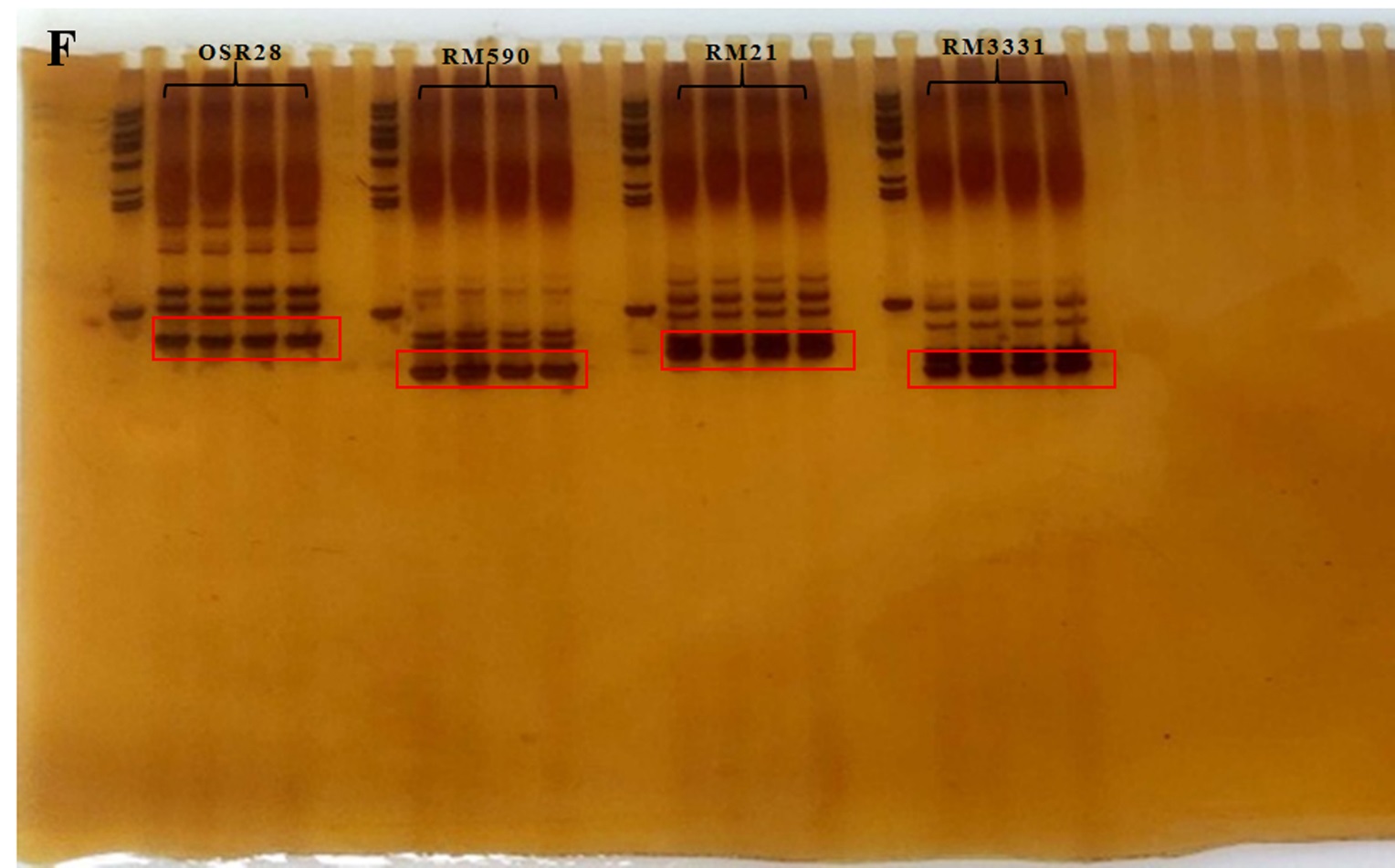


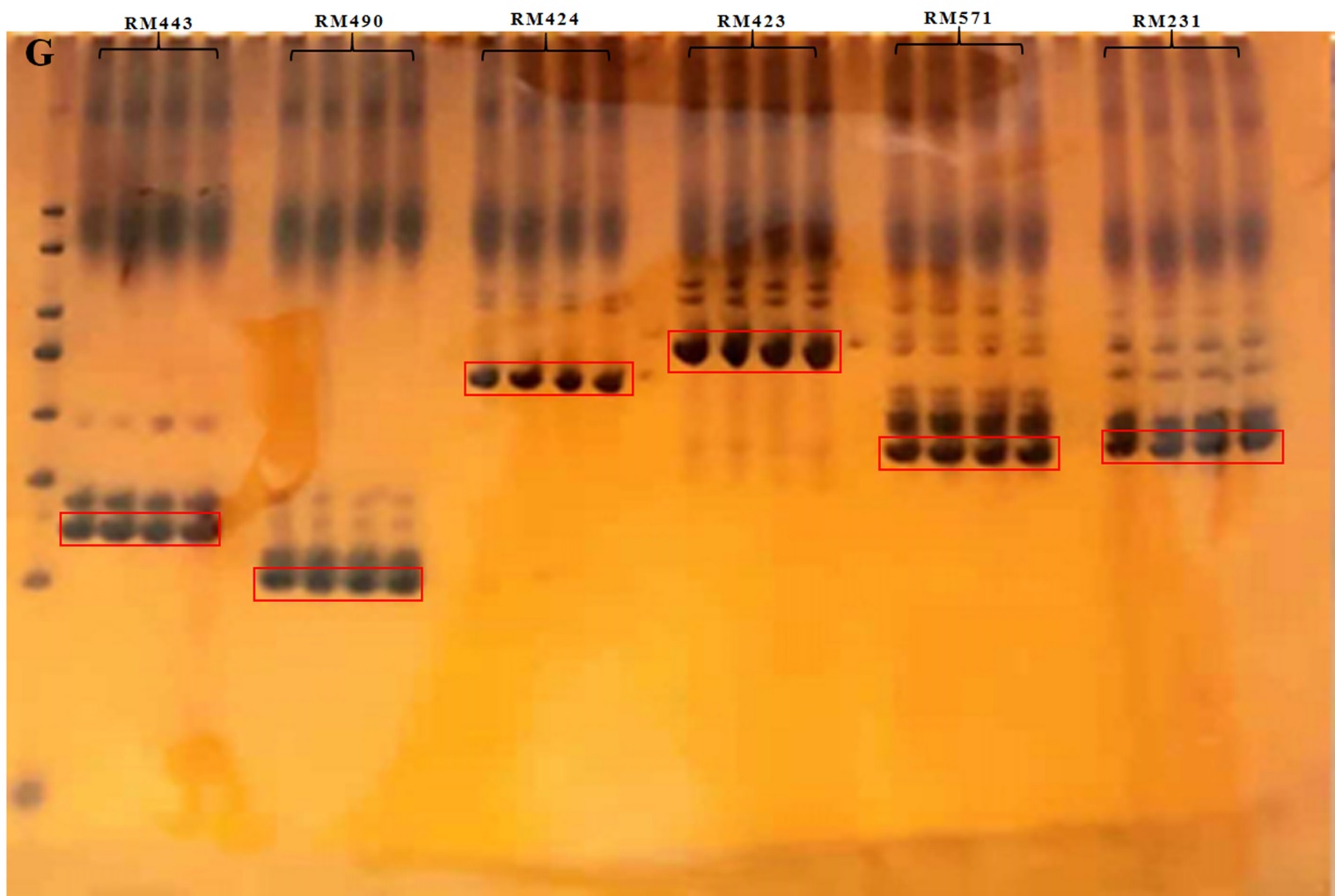


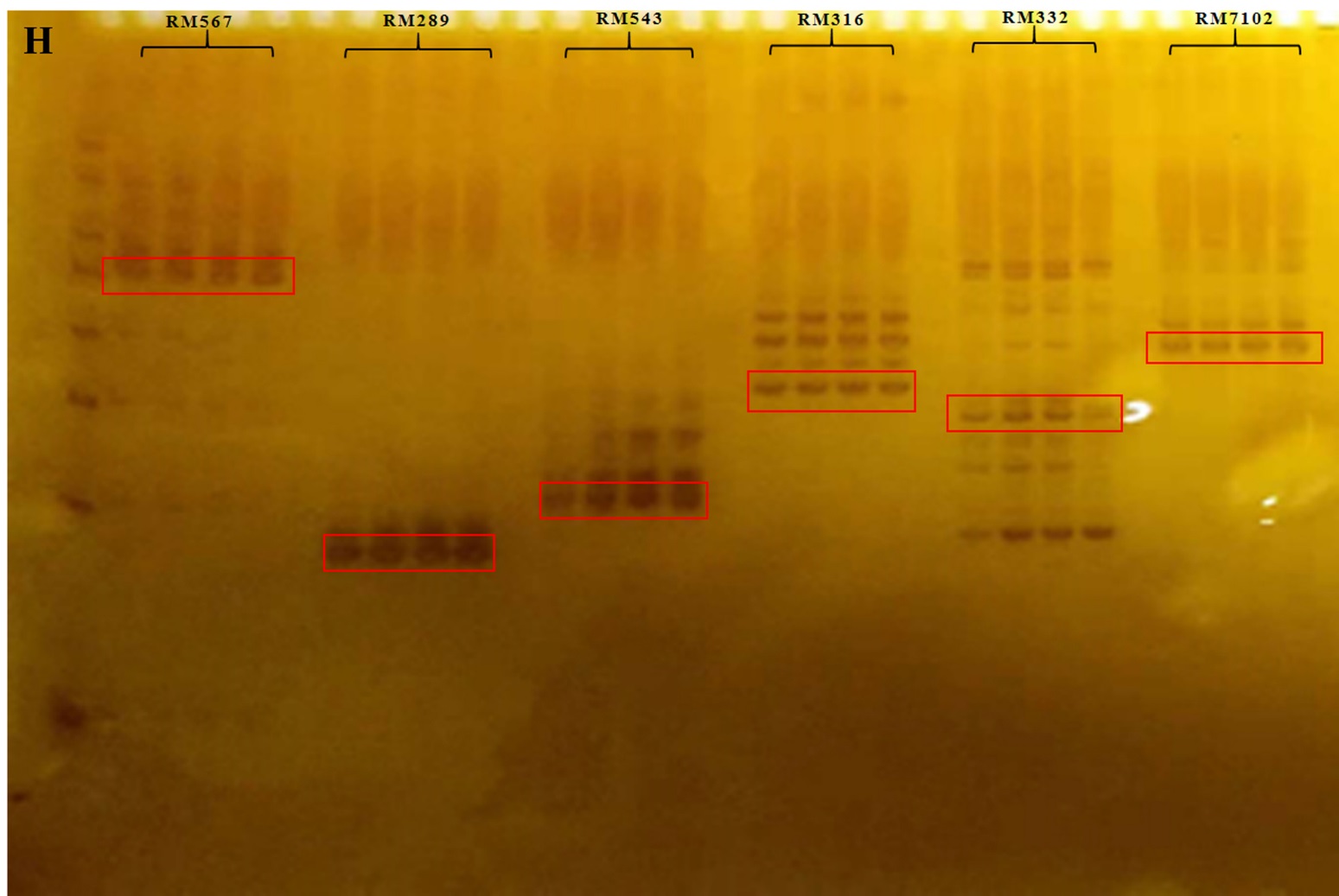

Supplement: Supplementary file 1 — Supplementary Information. [file 41598_2023_33742_MOESM1_ESM.docx]
